# The Effects of *Bacillus licheniformis*—Fermented Products on the Microbiota and Clinical Presentation of Cats with Chronic Diarrhea

**DOI:** 10.3390/ani12172187

**Published:** 2022-08-25

**Authors:** Ting-Wei Lee, Tzu-Yi Chao, Hui-Wen Chang, Yeong-Hsiang Cheng, Ching-Ho Wu, Yen-Chen Chang

**Affiliations:** 1 Graduate Institute of Molecular and Comparative Pathobiology, School of Veterinary Medicine, National Taiwan University, No. 1, Section 4, Roosevelt Rd., Taipei 106216, Taiwan; 2 Institute of Veterinary Clinical Science, School of Veterinary Medicine, National Taiwan University, Taipei 10617, Taiwan; 3 Department of Biotechnology and Animal Science, National Ilan University, No. 1, Section 1, Shennong Rd., Yilan City 260007, Taiwan

**Keywords:** *Bacillus licheniformis*-fermented products, probiotics, fecal microbiota, chronic feline diarrhea, next-generation sequencing

## Abstract

**Simple Summary:**

Probiotics are defined as live microorganisms that, when administered in adequate amounts, confer a health benefit to the host. Probiotics have also been introduced in companion animals in recent years, and the main priority of probiotic use has focused on the health of individual animals. This study aimed to compare the fecal microbiota of cats with chronic diarrhea with that of healthy cats from the same household and evaluate the effectiveness of oral administration of *Bacillus licheniformis*-fermented products (BLFP) in relieving clinical signs and altering the intestinal microbiota in diarrheal cats. Overall, this study provides basic information on the types and ratios of fecal microbiota in healthy cats and cats with chronic diarrhea. Our results also demonstrated that oral administration of BLFP could significantly impact the relief of clinical gastrointestinal symptoms and the gastrointestinal microbiomes in cats with diarrhea.

**Abstract:**

*Bacillus licheniformis*-fermented products (BLFP) are probiotics with antibacterial, antiviral, and anti-inflammatory properties that can improve growth performance. This study aimed to compare the fecal microbiota of diarrheal cats with chronic diarrhea (*n* = 8) with that of healthy cats (*n* = 4) from the same household using next-generation sequencing, and evaluate the effectiveness of oral administration of BLFP in relieving clinical signs and altering the intestinal microbiota in diarrheal cats. Six out of eight diarrheal cats showed clinical improvement after BLFP administration for 7 days, and the stool condition of the other two was normal. A higher *Firmicutes*/*Bacteroidetes* ratio was noted in the feces of diarrheal cats without clinical improvement as compared with those in the healthy cats and in the diarrheal cats with clinical improvement after receiving BLFP. The phylum *Bacteroidetes* and class *Bacteroidia* decreased significantly in diarrheal cats regardless of BLFP administration. *Blautia* spp., *Ruminococcus torques*, and *Ruminococcus gnavus,* which belong to the *Clostridium* cluster XIVa and have been reported as beneficial to intestinal health, increased significantly in feces after treatment. Furthermore, *Clostridium perfringens* also significantly decreased in diarrheal cats after BLFP administration. Overall, BLFP could be a potential probiotic to relieve gastrointestinal symptoms and improve fecal microbiota in cats with chronic diarrhea.

## 1. Background

According to the World Health Organization [1], probiotics are defined as live microorganisms that, when administered in adequate amounts, confer a health benefit to the host. For decades, probiotics have been used in farm animals to promote economic efficiency by improving feed conversion, increasing production, promoting growth, protecting the host from enteropathogens, and stimulating immune responses [2]. Probiotics have also been introduced in companion animals in recent years, and the main priority of probiotic use has focused on the health of individual animals [3]. The effects of probiotics on dogs and cats have been evaluated, including *Enterococcus faecium* SF68 in shelter cats and dogs with diarrhea [4], *Bifidobacterium animalis* in dogs with acute idiopathic diarrhea [5], and *Lactobacilli* isolated from canine milk [6].

*Bacillus* species have been broadly used in human medicine with applications as enzyme, amino acid, and antibiotic production systems; preparation of fermented foods; and pest control [7]. Several *Bacillus* species, including *Bacillus clausii*, *B. coagulans*, *B. subtilis*, *B. licheniformis*, and *B. cereus*, have been shown to possess immune stimulation and antimicrobial activities and were competitive exclusion agents in humans [8]. *Bacillus licheniformis*-fermented products (BLFP) applied in the present study is obtained from solid-state fermentation (SFF) and contains spores, stable in the environment as a favored biomass form for probiotics, and secondary metabolites, such as biosurfactants. SFF has a high yield and requires inexpensive equipment, and thus has been widely applied for scaled-up production [9]. In veterinary medicine, using BLFP as dietary supplementation has improved the growth performance of broilers [10], mitigated necrotic lesions caused by *Clostridium perfringens* infection in poultry [9], and alleviated clinical syndromes with reduced viral loads in porcine epidemic diarrhea virus (PEDV)-infected pigs [11]. However, the effects of *B. licheniformis*-fermented products on companion animals have not yet been evaluated.

Humans and animals have complex and vast microbial communities inhabiting the gastrointestinal tract which serve to maintain homeostasis, support nutrient absorption and metabolism, and regulate the inflammatory immune response [12,13,14]. Therefore, alteration of bacterial flora in the gastrointestinal tract will lead to dysbiosis, which, according to Marsilio and others [15], has three main hallmarks: reduction of bacterial diversity, higher fluctuation rate due to decreased stability of microbiota, and reduction of obligate anaerobic flora with an increase in facultative anaerobes. Dysbiosis may lead to gastrointestinal disturbances and is associated with diseases such as atherosclerosis [16], ulcerative colitis [17], Crohn’s disease [18], and colorectal cancer [19] in humans.

Animals have a distinct intestinal microbiota composition, which varies among animal species, gut niches, and geographic regions [14]. In felines, the major bacterial phyla in the gastrointestinal microbiota are *Firmicutes*, *Bacteroidetes*, *Actinobacteria*, and *Proteobacteria*, similar to those in other mammals, including canines and humans [19]. Bermingham and others found that the predominant genera in kittens (18 weeks old) were *Lactobacillus* and *Bifidobacterium*, while the dominance changed to *Bacteroides*, *Prevotella*, and *Megasphaera* in 42-week-old cats, indicating that the fecal microbiota is affected by age in cats [20]. A study showed a significant increase in bacterial groups including the order *Burkholderiales*, the family *Enterobacteriaceae*, and the genera *Streptococcus* and *Collinsella* in diarrheal cats. In healthy cats, the order *Campylobacterales*, the family *Bacteroidaceae*, and the genera *Megamonas*, *Helicobacter*, and *Roseburia* were significantly increased compared to their presence in diarrheal cats. In acute diarrheal cats, the class *Erysipelotrichia* and the genus *Lactobacillus* were significantly decreased, and in chronic diarrheal cats, the phylum *Bacteroidetes* was significantly decreased compared to that in healthy cats [21]. In another study, the phyla *Firmicutes* and *Bacteroidetes* decreased and *Proteobacteria* increased in cats suffering from inflammatory bowel disease, along with the finding of lower diversity of *Clostridium* clusters XIVa and IV [22]. Marsilio and others found that alpha diversity indices were significantly decreased in cats with chronic enteropathy, including inflammatory bowel disease and small-cell lymphoma [15]. Furthermore, the linear discriminant analysis effect size (LEfSe) results showed that the families *Ruminococcaceae* and *Turicibacteraceae*, genus *Bifidobacterium*, and *Bacteroides plebeius* were lower, while the families *Enterobacteriaceae* and *Streptococcaceae* were higher in cats with chronic enteropathy than in normal cats. The order *Lactobacillales*, family *Enterococcaceae*, and genera *Enterococcus* and *Dorea* were increased in cats with *Toxocara cati* infestation in a study on the relationship between nematode infestation and intestinal microbiota [22].

This study aimed to compare the types and ratios of fecal microbiota inhabiting healthy cats with those of cats exhibiting intermittent diarrhea (stool score > 5) for over three weeks from the same household. Feces of these cats were collected, and their microbiomes were analyzed using next-generation sequencing (NGS). After establishing the baseline of the microbiomes in non-diarrheal and diarrheal cats, the effects of oral administration of BLFP on relieving the clinical signs and alteration of microbiomes in cats with diarrhea were evaluated.

## 2. Methods

### 2.1. Animals and Treatments

Twelve cats from the same household were included in this study and their signalments were provided in Table 1. All the cats were fed a similar diet and had the same exercise level. The health status of these cats was observed and evaluated by the owner and veterinarians. To evaluate the effects of BLFP on relieving the clinical signs and alteration of the microbiomes in cats with diarrhea, and to ensure that each cat received an equal amount of BLFP intake, 1.1 mg/kg BLFP was prepared as previously described [11] and supplied in a capsule that was manually fed to the cats by the owner once a day for 7 consecutive days. Clinical scoring and fecal samples of these cats were collected before and after 7 days of BLFP treatment.

### 2.2. Stool Scoring

The owner performed stool scoring based on the Bristol stool scoring system [23]. Seven stool statuses were classified. Type 1 was very hard and dry with separate hard lumps; type 2 was firm but not hard with a segmented appearance; type 3 was sausage-shaped with little or no segmentation and may have a moist surface; type 4 was smooth and very moist; type 5 was very soft but still had a distinct edge and formed a pile; type 6 was a mushy consistency with no defined shape; and type 7 was watery without solid pieces. An average score was selected if the owner could not distinguish between the two types. Types 1 and 2 were considered constipation; types 3 and 4 were normal feces; and types 5 to 7 were diarrheal stools. All feline fecal samples were scored before and after oral BLFP treatment.

### 2.3. Feline Chronic Enteropathy Activity Index (FCEAI)

Based on the established FCEAI [24], the clinical signs, including activity, appetite, vomiting, diarrhea, and weight loss, were scored as 0 = normal, 1 = mild, 2 = moderate, and 3 = severe by the owner in the designed questionnaire. The results of blood tests, including total protein, alanine aminotransferase (ALT), alkaline phosphatase (ALP), and phosphorus, were evaluated by a veterinary clinician and scored as 0 = normal and 1 = abnormal. The sum of these scores was calculated as the FCEAI to evaluate the health status of the animals. This study did not use endoscopic evaluation because of the owner’s unwillingness.

### 2.4. Fecal Collection

Feces were collected before and after 7 days of oral administration of BLFP. All fecal samples were freshly collected after defecation by the cats and maintained at ambient temperature until arrival at the laboratory. The samples were then manually homogenized and stored at −20 °C.

### 2.5. DNA Extraction

For each sample, 200 mg of the homogenized fecal samples were used for DNA extraction using a commercial stool extraction kit (QIAamp Fast DNA Stool Mini Kit, Qiagen, Hilden, Germany), following the manufacturer’s instructions.

### 2.6. Next-Generation Sequencing

Quantitation, sequencing, and analysis of the microbiome were performed by Tri-I Biotech Incorporation (New Taipei City, Taiwan). DNA quantity and concentration were measured using the Qubit Fluorometer (Thermo Fisher Scientific Inc., Waltham, MA, USA). The V3-V4 region of the bacterial 16S rRNA was amplified using primer sets 341F and 805R and Kapa Biosystems (Wilmington, NC, USA). The PCR conditions were 95 °C for 3 min; 25 cycles of 98 °C for 20 s, 57.5 °C for 20 s, and 72 °C for 20 s; and a final extension step at 72 °C for 3 min. Six 8-based barcoded sequences were added to the forward primer to multiplex the amplicon pools. After pooling the amplicons, the PCR products were purified and quantified using the QIAquick PCR Purification Kit and cleaned with AMPure XP beads (Beckman Coulter Inc., Brea, CA, USA).

### 2.7. Library Construction and Sequencing

The Celero DNA Seq Library Preparation Kit (NuGEN) was used for whole-genome sequencing and library construction, followed by 2 × 301 bp paired-end sequencing using the MiSeq system. The raw sequencing reads were merged, filtered, and generated to obtain effective reads. Assembled reads were assigned to the corresponding samples using the default parameters. Thereafter, reads shorter than 293 bp or longer than 455 bp were removed, and 95% of the reads were retained. All reads were then aligned using NAST-based sequence alignment based on SILVA alignment. Reads that did not align with the anticipated region of the reference alignment and chimeric sequences identified using the UCHIME algorithm were removed. All reads were classified using a Bayesian classifier in the Silva database.

### 2.8. Statistical Analyses

All the effective reads from all samples were clustered into OTUs based on 97% sequence similarity using USEARCH. The OTUs were then classified based on the taxonomy of the reads. Subsampling was performed to normalize the sequences. To estimate richness, evenness, and diversity, the rarefaction curves of the Chao1, Simpson, and Shannon indices were computed and drawn using QIIME. Principal coordinate analysis (PCoA) was used to determine beta diversity, which adopted two metrics: unweighted UniFrac and weighted UniFrac. Taxonomic profiling of samples at different taxonomic levels (class, order, family, genus, and species) and corresponding bar plots were created using QIIME. Statistical significance was determined by Metastats and two-way ANOVA (PRISM, GraphPad software Inc., San Diego, CA, USA). Statistical significance was set at *p* < 0.05. LEfSe was performed.

## 3. Results

### 3.1. Animals and Grouping

Twelve cats aged 3 to 15 years from the same household were enrolled in this study. Five of them were spayed females, and the rest were castrated males. Ten cats were domestic shorthair (DSH) cats, one was a Scottish fold, and one was an American shorthair. No medication history was reported for any cat one month before and during the study period. The results of the FCEAI and stool scoring before and after the BLFP are shown in Table 1. The four cats that exhibited normal stool scores were not treated with BLFP.

### 3.2. Stool Scoring

Based on the Bristol stool scoring system, the cats were scored at 2.5–7 before BLFP treatment. Eight cats (cats 1–8), scoring from 5–7, were defined as diarrheal cats and included in the treatment group. The other four cats (cats 9–12) had normal stools with a score of 2.5–4 and were defined as normal cats and included in the non-diarrhea control group (Table 1).

All cats in the treatment group showing intermittent diarrhea (cats 1–8) were orally treated with BLFP every day for 7 consecutive days. Before the BLFP treatment, 3/8 cats were scored at 5, 3/8 were scored at 5.5, and 2/8 were scored at 7 (Table 1). All were considered to have diarrhea. After the 7-day treatment with BLFP, 6/8 cats (cats 1, 2, 3, 6, 7, and 8) showed improvement in fecal status. The feces of 2/6 cats (cats 1 and 2) were normal in shape, and both were scored at 2.5. During the study, no alteration in fecal score was noted in the control group (Table 1).

### 3.3. Feline Chronic Enteropathy Activity Index (FCEAI)

The FCEAI results varied for all groups. An additional file shows this in more detail (Table S1). In the control group, the FCEAI ranged from 1–4 and showed minimal differences from those of the BLFP treatment group, ranging from 1–5. In the control group, three cats vomited, and two showed weight loss, but none had diarrhea. In the blood examination, two cats had elevated total protein levels, while the rest of the animals exhibited normal FCEAI.

In the treatment group (*n* = 8), all cats showed variable diarrhea conditions (scored 1–3), four cats vomited, and two cats had a mild increase in alanine aminotransferase prior to the BLFP treatment. After the treatment, four cats showed improvement in diarrhea status, and both cats showed reduced vomiting. The total FCEAI score in the BLFP treatment group reduced from 1–5 to 1–4. The FCEAI score decreased in three cats, while the other five cats had the same score after 7 days of BLFP treatment. An additional file shows this in more detail (Table S1).

### 3.4. Next Generation Sequencing (NGS)

#### 3.4.1. Sequence Analysis and Rarefaction

**Figure 1:**
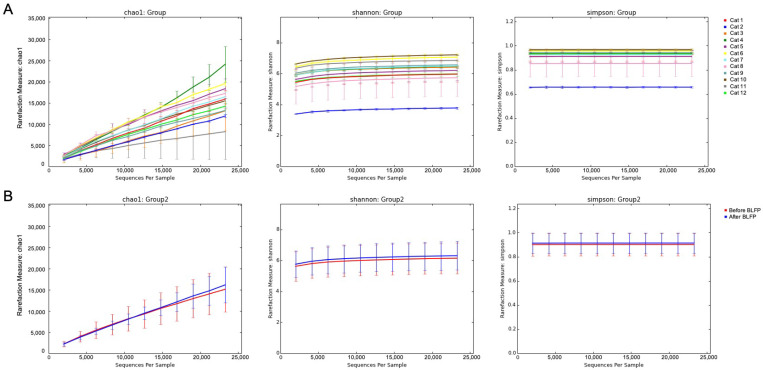
Alpha diversity indices, including Chao1, Shannon, and Simpson. (**A**): All of the samples. (**B**): Alpha diversity of microbial communities before (red) and after (blue) the treatment of *Bacillus licheniformis*-fermented products (BLFP) in the diarrheal cats.

**Figure 2:**
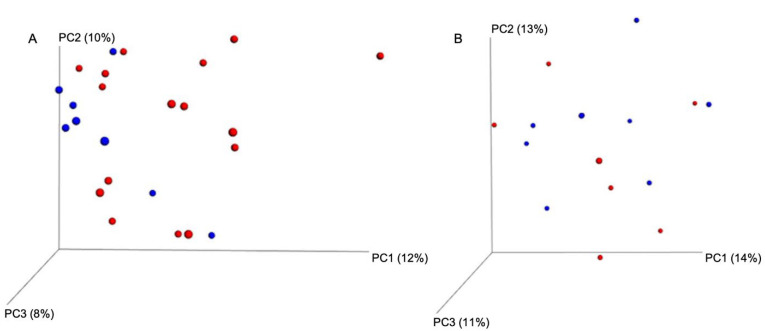
Beta diversity based on principal coordinate analysis (PCoA). (**A**): diarrheal cats (red dots) and control cats (blue dots). (**B**): diarrheal cats before (red dots) and after (blue dots) the treatment of *Bacillus licheniformis*-fermented products (BLFP).

A total of 1,285,308 effective read sequences, with an average of 53,555 sequences in pairs/samples (ranging from 51,596–56,752), were retained from the 24 fecal samples obtained from the four cats in the control group and eight cats in the BLFP treatment group before and 7 days after BLFP treatment. The fecal microbiome patterns in these samples were variable but overall were high in richness and diversity using the Chao1, Shannon, and Simpson indices (Figure 1A). Regardless of the breed, gender and age, no significant difference in diversity was detected before or after BLFP treatment. There was a slightly higher richness and diversity in the fecal microbiome from the diarrheal cats after BLFP administration than before the treatment (Figure 1B). However, no statistically significant difference was noted. According to the beta diversity shown in the PCoA, the fecal microbiomes of the samples were not significantly grouped in either the treatment or control groups or in the treatment groups before and after BLFP administration (Figure 2). However, the treatment group was relatively more closely grouped than the control group.

#### 3.4.2. Operational Taxonomic Unit (OTU)-Based Analysis

The sequences of the fecal microbiome were clustered into 53,157 OTUs, with an average of 3123 OTUs in each sample (ranging from 1520 to 3872). OTUs were classified into 38 phyla, 87 classes, 147 orders, 236 families, 593 genera, 985 species, and other unclassified reads.

**Figure 3:**
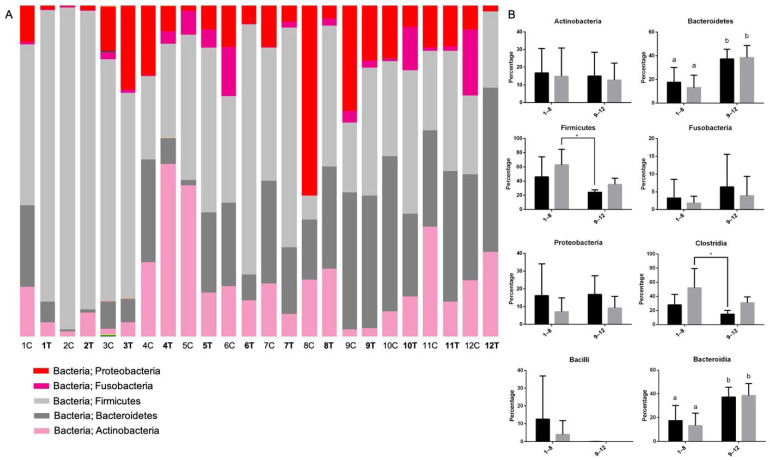
(**A**) Relative distribution of the bacterial phyla of the fecal microbiome in the eight cats (No. 1–8) of the treatment group and the four cats (No. 9–12) of the control group before and after the treatment of *Bacillus licheniformis*-fermented products (BLFP). (**B**) Comparisons of the dominant phyla and class component between in the treatment (No. 1–8) group before and after the treatment with BLFP and in the control group (No. 9–12) without BLFP treatment. Black bar: before having BLFP in the treatment group or first sampling of feces in the control group. Grey bar: after having BLFP in the treatment group or second sampling of feces in the control group. * indicates *p* < 0.05.

A total of 38 bacterial phyla were detected, with the most dominant being *Firmicutes* (46%), *Bacteroidetes* (23%), *Actinobacteria* (15%), *Proteobacteria* (12%), and *Fusobacteria* (3%), accounting for 98% of the total OTUs in each sample. The samples’ relative distribution, a bar chart of average percentage, a comparison before and after BLFP administration, and the percentage breakdown of the bacterial phyla are presented in Figure 3 and Table S2. The dominant bacterial phyla in the control group were *Bacteroidetes* (37%), *Firmicutes* (24%), *Proteobacteria* (17%), *Actinobacteria* (15%), and *Fusobacteria* (6%) (Figure 3A). However, *Firmicutes* (46%) was more dominant in the treatment group, followed by *Bacteroidetes* (18%), *Actinobacteria* (17%), *Proteobacteria* (16%), and *Fusobacteria* (3%) (Figure 3B). There were a higher proportion of *Firmicutes* and a lower proportion of *Bacteroidetes* in the treatment group than in the control group (Figure 3B). There was a significantly decreased proportion of *Bacteroidetes* in the treatment group compared to the control group. After the BLFP treatment, increased *Firmicutes* (63%) and decreased *Proteobacteria* (7%) levels were observed (Figure 3B).

**Figure 4:**
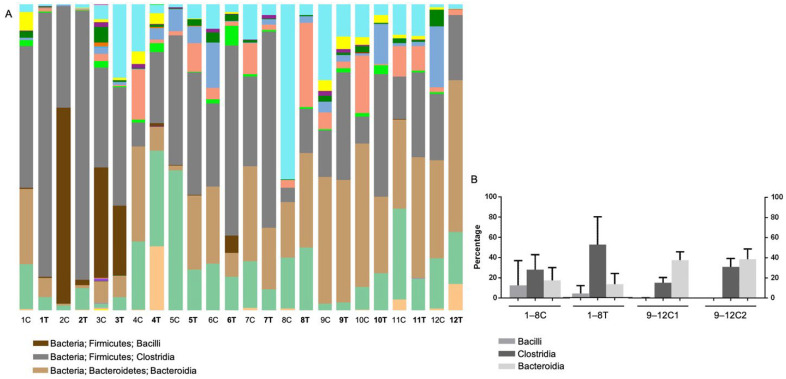
(**A**) Relative distribution of the bacterial classes in fecal microbiome of eight cats (1–8) from the treatment group and four cats (9–12) from the control group before and after the *Bacillus licheniformis*-fermented products (BLFP) treatment. The list of the represented bacterial classes of other color bars is provided in Figure S1. (**B**) Bar chart of the selected bacterial class components in cats having diarrhea before (1–8C) and after (1–8T) BLFP treatment and in non-diarrheal cats without having BLFP (9–12C1 and C2). C: before the BLFP treatment; T: after the BLFP treatment; C1: first sampling of feces in the control group; C2: second sampling of feces in the control group.

A total of 87 classes were identified in all the OTUs. The relative distribution, bar chart of average percentage, and the detailed percentage of the bacterial classes are shown in Figure 4 and Table S3, respectively. The main classes included *Actinobacteria*, *Coriobacteriia*, *Bacteroidia*, *Bacilli*, *Clostridia*, *Erysipelotrichia*, *Negativicutes*, *Fusobacteriia*, *Betaproteobacteria*, *Deltaproteobacteria*, *Epsilonproteobacteria*, and *Gammaproteobacteria*, accounting for approximately 99% of the total classes (Table S3). The main classes in the control group were *Bacteroidia* (37.9%), *Clostridia* (22.9%), *Coriobacteriia* (12.2%), *Gammaproteobacteria* (9%), and *Fusobacteriia* (5.3%). In the treatment group, the composition of the bacterial classes was *Clostridia* (27.9%), *Bacteroidia* (17.4%), *Coriobacteriia* (16.6%), *Bacilli* (12.5%), and *Gammaproteobacteria* (12.5%). There was a significant increase in *Bacteroidia* in the control group compared to the treatment group, regardless of BLFP administration (Figure 4A,B). Two samples (Cats 2 and 3) in the treatment group showed a higher proportion of *Bacilli* (64.1% and 35.9%, respectively) before BLFP treatment. After the BLFP treatment, the prevalent taxa changed to *Clostridia* (52.1%), *Bacteroidia* (13.1%), *Coriobacteriia* (12.2%), *Gammaproteobacteria* (5.4%), *Negativicutes* (5.4%), and *Bacilli* (4%). Additionally, the proportion of *Bacilli* in Cat 2 and Cat 3 decreased to 1.7% and 22.7%, respectively. In Cat 4, the proportion of *Actinobacteria* in feces was relatively higher (20.9%) than that in other cats with diarrhea. Interestingly, an increased proportion of *Clostridia* in the feces was observed in seven cats after BLFP administration (Figure 4A).

**Figure 5:**
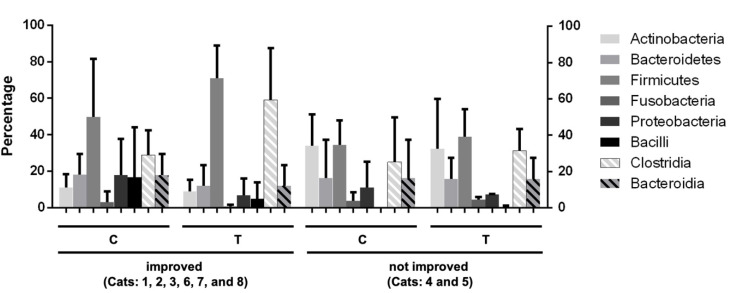
Changes in the percentage of selected bacterial phylum and class components in diarrheal cats having clinical improvement (cats: 1, 2, 3, 6, 7, and 8) before and after *Bacillus licheniformis*-fermented products (BLFP) treatment and diarrheal cats (cats: 4 and 5) without clinical improvement. C: before the BLFP treatment; T: after the BLFP treatment.

The phylum *Firmicutes* and class *Clostridia* had a higher proportion after BLFP treatment in diarrheal cats that showed clinical improvement (cats 1, 2, 3, 6, 7, and 8) (Figure 5). However, the difference was not significant. In contrast, diarrheal cats without clinical improvement showed minimal changes in the gut microbiota, as shown in Figure 5. The alterations in the fecal microbiome with significant differences (*p* < 0.05) by Metastats analysis before and after BLFP administration in cats with diarrhea are shown in Table 2. According to the results, 10/14 samples showed significant enrichment, of which 5/10 belonged to the order *Clostridiales,* and 5/10 were in the order *Lactobacillales*. The most abundant OTUs were *Lactococcus garvieae*, *Ruminococcus gnavus*, *Clostridium*_uc (unclassified genus), *Blautia*uc, *Paeniclostridium*_uc, *Ruminococcus torques*, *Anaerotruncus*_uc, and *Enterococcus durans*. A few OTUs showed a significant decrease, including *Acidaminococcus*_uc, *Butyricicoccus*_uc, *Bacteroides dorei*, *Clostridium perfringens*.

#### 3.4.3. Results of Linear Discriminant Analysis Effective Size (LEfSe)

**Figure 6:**
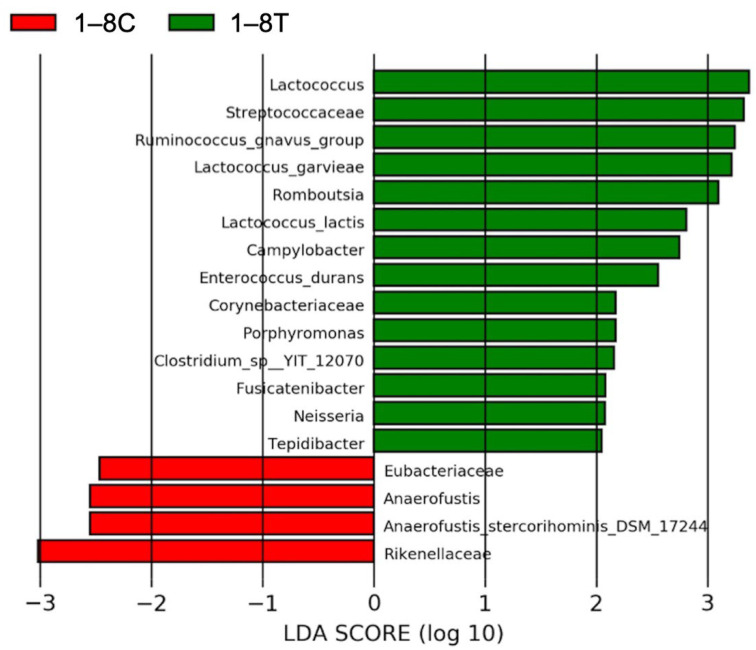
The linear discriminant analysis (LDA) scores based on linear discriminant analysis effect size (LEfSe) results before (red) and after (green) *Bacillus licheniformis*-fermented products (BLFP) administration in diarrheal cats. C: before the BLFP treatment; T: after the BLFP treatment.

To compare the fecal microbiome before and after BLFP administration, linear discriminant analysis (LDA) scores based on the LEfSe results were calculated (Figure 6). The results showed that 14 taxonomic units were enriched, and 4 decreased after BLFP administration in the treatment group. Among the enriched OTUs, 11/14 belonged to the phylum *Firmicutes*, 5/14 to the order *Clostridiales*, and 5/14 to the order *Lactobacillales*. The 14 most abundant taxonomic units were *Lactococcus*, *Sreptococcaceae*, *Ruminococcus gnavus*, *Lactococcus garvieae*, *Romboutsia*, *Lactococcus lactis*, *Campylobacter*, *Enterococcus durans, Corynebacteriaceae*, *Porphyromonas*, *Clostridium* sp., *Fusicatenibacter*, *Neisseria*, and *Tepidibacter*. The four OTUs with the lowest abundance were *Eubacteriaceae*, *Anaerofustis, Anaerofustis stercorihominis*, and *Rilenellacea.*

## 4. Discussion

Chronic diarrhea is common in cats, and its prevalence is high in multi-cat households. In this study, cats from the same multi-cat household were enrolled to ensure consistency of the environment and feeding conditions with or without BLFP treatment. Compared with non-diarrheal cats, diarrheal cats had a higher proportion of *Firmicutes* and a lower proportion of *Bacteroidetes*. After BLFP administration in diarrheal cats, there was a significant enrichment in the *Clostridiales* and *Lactobacillales* orders using Metastats analysis and a significant decrease in *Clostridium perfringens* by OTU analysis. This study provides basic information on the types and ratios of fecal microbiota in healthy cats and cats with chronic diarrhea. Our results also demonstrated that oral administration of BLFP could significantly impact the relief of clinical gastrointestinal (GI) symptoms and the GI microbiomes in cats with diarrhea.

After oral administration of BLFP, most diarrheal cats (6/8) showed noticeable improvement in stool condition, and some (3/8) exhibited a decreased FCEAI score. The age of the six diarrheal cats with improvement after BLFP ranged from 3–12 years. Among the six cats with improved diarrhea, the stool condition in two cats became normal. These results suggest that BLFP has a positive effect on improving chronic feline diarrhea. In several studies, BLFP has been demonstrated to improve growth performance, mitigate bacterial infection, and reduce the viral load in farm animals [9,10,11]. *Bacillus* has antagonistic activity against various bacteria by producing bacteriocins [25] and lipopeptides, such as bacillomycin L, plipastatin, and surfactin, to solubilize the bacterial membrane [26]. Regarding the antiviral activity of *Bacillus*, surfactin derived from BLFP has been suggested to compete for the entry receptor with transmissible gastroenteritis virus (TGEV) [27]. *Bacillus* can also produce extracellular polysaccharides to inhibit pro-inflammatory mediators and mitigate inflammation [28]. The above antibacterial, antiviral, and anti-inflammatory properties may have contributed to improving diarrhea in the current study. However, the actual mechanism should be further investigated.

In the present study, the major components of the fecal microbiota were the phyla *Firmicutes*, *Bacteroidetes*, *Actinobacteria*, *Proteobacteria*, and *Fusobacteria*, covering 98% of the total OTUs in both diarrheal and non-diarrheal cats. This is similar to previous findings in felines with variable proportions [29]. Comparing the proportions of various phyla and classes, only *Bacteroidetes* and *Bacteroidia* showed a significantly lower proportion in diarrheal cats than in healthy cats, regardless of BLFP administration. Decreased *Bacteroidetes* abundance in GI disturbance has been frequently reported in cats, dogs, and humans [30,31,32,33]. Although *Firmicutes* were decreased in human patients with inflammatory bowel disease [34] and dogs with IBD [35], the increased *Firmicutes* observed in the current study is consistent with that reported in diarrheal cats [36]. This indicates that changes in the microbiome under diarrheal conditions may differ among species. Although no statistical difference was evaluated by comparing the richness and diversity between diarrheal and non-diarrheal cats using alpha diversity indices, trends that the diarrheal cats were grouped more closely than the healthy cats were observed. The overall microbiota composition may differ between cats with GI disturbances and healthy cats, which is similar to the result of a study comparing diarrheal and normal cats [36]. The tendency of a relatively higher proportion of *Firmicutes* and lower *Bacteroidetes* after BLFP administration in diarrheal cats in the present study is similar to a previous study in broilers using the same BLFP product [10]. There was a slightly higher richness and diversity of the microbiota in alpha diversity indices in the diarrheal cats after BLFP administration than before BLFP treatment, while no statistical significance was observed.

The metastats analysis of OTU-based microbiome and LEfSe showed that *R. gnavus*, *L. garvieae*, *L. lactis* and *E. durans* were significantly higher in the feces of BLFP-treated cats. *R. gnavus* was associated with inflammatory bowel disease by producing inflammatory polysaccharides in humans [35]. However, there is a lack of information regarding whether *R. gnavus* plays a similar role in animals. Since the diarrhea condition of the BLFP-treated cats was improved rather than worsened in this study, the role of increased *R. gnavus* in the intestinal microenvironment should be further investigated. Generally, common lactic acid-producing bacteria which are considered to provide beneficial effect on improving gastrointestinal diseases in dogs and cats, such as *Lactobacillus*, *Enterococcus*, *Streptococcus*, and certain strains of *Bifidobacterium* species [37], displayed no statistical significances before and after BLFP administration in the diarrheal cats. Although *L. garvieae* and *L. lactic* are capable of producing lactic acid during fermentation and not pathogenic for humans and could produce antimicrobial substance [38], a few studies have evaluated the function in the intestinal microbiome communities in veterinary medicine. There are more studies on marine life because *L. garviae* is an important pathogen in the ocean; for example, a significantly higher population of *L. garviae* was reported in sea cucumbers with skin ulceration syndrome than in healthy individuals [39]. As for *L. lactis*, several published studies indicated that certain strains of *L. lactis* as probiotic feed additive could modulate intestinal immunity and microbiota and thus prevent intestinal pathogen infection in piglets and fishes [40,41]. *E. durans* has been demonstrated to increase after an elemental diet, a nutritional therapy for Crohn’s disease, in a mouse model of chronic colitis [42].

A decrease in *C. perfringens* was noted in cats with diarrhea after BLFP administration in this study. An increase in the density of *C. perfringens* has been reported in animals and humans with diarrhea [43,44,45]. An in vitro study has demonstrated the potential of probiotics such as *Lactobacillus plantarum*, *Lactobacillus rhamnosus*, and *Bifidobacterium animalis* lactis strains to inhibit *C. perfringens* [46]. The density of *C. perfringens* is believed to play an important role in intestinal dysbiosis. Toxin-producing *C. perfringens* is associated with acute hemorrhagic diarrhea syndrome [47]. Molecular detection of the enterotoxin gene (*cpe*) is required to confirm the pathogenicity of *C. perfringens*. Although a previous study reported that the amount of *C. perfringens* had no impact in cats with GI disease [36], *C. perfringens* was detected in cats with diarrhea and could be reduced and controlled by BLFP administration in this study. *Blautia* spp., *R. torques*, and *R. gnavus* belong to the clostridial subcluster XIVa, producing short-chain fatty acids (SCFA) to maintain intestinal health. A significant decrease in the density of *Blautia* spp. has been reported in dogs with acute hemorrhagic diarrhea [44]. A significant increase in *Blautia* spp. *R. torques* and *R. gnavus* were observed in diarrheal cats after BLFP administration, suggesting an improvement in gut microbiome communities.

Only one household was enrolled in this study. This eliminated most of the variables, such as different environments, diets, exercise levels, and breeds. However, further studies that include those variables are needed. According to the results of fecal scoring, eight cats with a history of chronic diarrhea were included in the treatment group. Unfortunately, we could not determine the actual causes of diarrhea in each animal because examinations involving invasive procedures, such as biopsy and endoscopy, were refused by the owner. Intestinal parasitic infestation and apparent systemic infection were preliminarily ruled out using fecal flotation/microscopic analysis and clinical physical/blood examinations. Because the variability of microbiota composition may result from different geographic regions, e.g., diets, medications, ages, and GI disturbances [12,48,49], in this study comparisons of animals with different clinical signs and responses to treatments were performed in a single household with the same dietary intake and similar activity conditions to reduce the variation.

## 5. Conclusions

Although there is limited information on the specific microbiome and the mechanism maintaining intestinal homeostasis in cats to date, BLFP is a potential probiotic that improves the diarrheal condition clinically with proof of some positive impact on the intestinal microbiome communities, such as a significant increase of beneficial bacterial species (*Clostridium* cluster XIVa) and a significant decrease of *Clostridium perfringens*. However, further studies on the correlation between the observed phylogenetic differences in the intestinal microbiome communities and their function in gut homeostasis are still needed.

## Figures and Tables

**Table 1:** The list of the history, stool score, and the Feline Chronic Enteropathy Activity Index (FCEAI) in cats with diarrhea (1–8) and without diarrhea (9–12) before and after the treatment of *Bacillus licheniformis*-fermented products.

Number	Breed	Age	Gender	Stool Score		FCEAI	
				Before	After	Before	After
1	DSH	12	Mc	5	2.5	2	1
2	DSH	12	Fsp	5	2.5	5	2
3	DSH	3	Mc	5.5	5	2	2
4	DSH	7	Mc	5.5	5.5	4	4
5	DSH	5	Mc	5	5	1	1
6	DSH	9	Fsp	7	6.5	5	3
7	DSH	3	Mc	5.5	5	2	2
8	Scottish fold	3	Fsp	7	6.5	4	4
9	DSH	3	Fsp	3	3	1	2
10	DSH	5	Mc	4	4	4	5
11	DSH	3	Mc	3	3	2	3
12	American short hair	15	Fsp	2.5	2.5	1	1

DSH, domestic short hair; Fsp, spayed female; Mc, castrated male.

**Table 2:** The significant (*p* < 0.05) changes in OTU-based microbiome in diarrheal cats before and after the treatment with *Bacillus licheniformis*-fermented products (BLFP).

OTU	Phylum	Class	Order	Family	Genus	*p* Value	Fold Change
204	Firmicutes	Bacilli	Lactobacillales	Streptococcaceae	Lactococcus	0.00359	188.630666
200	Firmicutes	Clostridia	Clostridiales	Lachnospiraceae	Ruminococcus_gnavus_group	0.0058	9.36044619
24	Firmicutes	Clostridia	Clostridiales	Clostridiaceae_1	Clostridium_sensu_stricto_1	0.01115	238.432716
158	Firmicutes	Clostridia	Clostridiales	Lachnospiraceae	Blautia	0.01427	7.98145294
3	Firmicutes	Clostridia	Clostridiales	Peptostreptococcaceae	Paeniclostridium	0.01847	14.0464061
163	Firmicutes	Clostridia	Clostridiales	Clostridiaceae_1	Clostridium_sensu_stricto_1	0.02056	1.60162436
92	Firmicutes	Negativicutes	Selenomonadales	Acidaminococcaceae	Acidaminococcus	0.02345	−3.434832
626	Firmicutes	Clostridia	Clostridiales	Ruminococcaceae	Butyricicoccus	0.02598	−10.838548
81	Firmicutes	Clostridia	Clostridiales	Lachnospiraceae	Ruminococcus_torques_group	0.03359	5.76459407
260	Firmicutes	Clostridia	Clostridiales	Ruminococcaceae	Anaerotruncus	0.034	22.2240326
13	Bacteroidetes	Bacteroidia	Bacteroidales	Bacteroidaceae	Bacteroides	0.03848	−11.225547
411	Firmicutes	Bacilli	Lactobacillales	Enterococcaceae	Enterococcus	0.04221	13.6340283
135	Firmicutes	Clostridia	Clostridiales	Ruminococcaceae	Ruminococcaceae_UCG-014	0.04417	46.5310365
636	Firmicutes	Clostridia	Clostridiales	Clostridiaceae_1	Clostridium_sensu_stricto_1	0.04948	−211.89874

## Data Availability

The datasets generated and analyzed during the current study are available at NCBI’s Gene Expression Omnibus (http://www.ncbi.nlm.nih.gov/geo/) under the accession GSE203254 (accessed on 21 August 2022).

## Supplementary Materials

The following supporting information can be downloaded at: https://www.mdpi.com/article/10.3390/ani12172187/s1, Table S1: The detailed criteria and scores for the Feline Chronic Enteropathy Activity Index (FCEAI) in each cat with diarrhea (No. 1–8) and without diarrhea (No. 9–12) before (C) and after (T) the treatment of *Bacillus licheniformis*-fermented products (BLFP); Table S2: The relative percentage of various bacterial phyla of fecal microbiome in the 8 cats (No. 1–8) of the diarrhea group and the 4 cats (No. 9–12) of the non-diarrhea group before and after the treatment of *Bacillus licheniformis*-fermented products (BLFP); Table S3: The relative percentage of various bacterial classes of fecal microbiome in the 8 cats (No. 1–8) of the diarrhea group and the 4 cats (No. 9–12) of the non-diarrhea group before and after the *Bacillus licheniformis*-fermented products (BLFP) treatment; Figure S1: The list of represented bacteria classes of other color bars in Figure 4.
