# Supplementary material for: The Effects of Bacillus licheniformis—Fermented Products on the Microbiota and Clinical Presentation of Cats with Chronic Diarrhea
Source: Animals (Basel). 2022 Aug 25;12(17):2187. doi: 10.3390/ani12172187 (PMC9454741; doi:10.3390/ani12172187)

Table S1. The detailed criteria and scores for the Feline Chronic Enteropathy Activity Index (FCEAI) in each cat with diarrhea (No. 1-8) and without diarrhea (No. 9-12) before (C) and after (T) the treatment of *Bacillus licheniformis*-fermented products (BLFP). All of the 4 non-diarrheal cats (No. 9-12) did not receive the BLFP treatment. C1: first sampling of feces in the control group; C2: second sampling of feces in the control group.

Number	Activity	Appetite	Vomiting	Diarrhea	Weight Loss	TP	ALT	ALP	Phos	Total
1C	0	0	1	1	0	0	0	0	0	2
1T	0	0	0	0	0	0	1	0	0	1
2C	0	0	2	1	2	0	0	0	0	5
2T	0	1	1	0	0	0	0	0	0	2
3C	0	0	0	2	0	0	0	0	0	2
3T	0	0	0	2	0	0	0	0	0	2
4C	0	0	1	2	1	0	0	0	0	4
4T	1	1	0	2	0	0	0	0	0	4
5C	0	0	0	1	0	0	0	0	0	1
5T	0	0	0	1	0	0	0	0	0	1
6C	0	0	2	3	0	0	0	0	0	5
6T	0	0	0	2	1	0	0	0	0	3
7C	0	0	0	2	0	0	0	0	0	2
7T	0	0	0	2	0	0	0	0	0	2
8C	0	0	0	3	0	0	1	0	0	4
8T	1	0	0	2	0	0	1	0	0	4
9C1	0	0	0	0	0	1	0	0	0	1
9C2	0	1	0	0	1	0	0	0	0	2
10C1	1	0	1	0	1	1	0	0	0	4
10C2	1	1	1	0	1	1	0	0	0	5
11C1	0	0	2	0	0	0	0	0	0	2
11C2	1	1	0	0	1	0	0	0	0	3
12C1	0	0	1	0	0	0	0	0	0	1
12C2	0	0	1	0	0	0	0	0	0	1

Table S2. The relative percentage of various bacterial phyla of fecal microbiome in the 8 cats (No. 1-8) of the diarrhea group and the 4 cats (No. 9-12) of the non-diarrhea group before and after the treatment of *Bacillus licheniformis*-fermented products (BLFP). C: before the BLFP treatment; T: after the BLFP treatment; C1: first sampling of feces in the control group; C2: second sampling of feces in the control group.

	Actinobacteria	*Bacteroidetes*	*Firmicutes*	*Fusobacteria*	*Proteobacteria*
1-C	15%	25%	48.6%	0.8%	10.9%
1-T	4.20%	6%	88%	0.30%	1.10%
2-C	1.50%	1%	97.30%	0.30%	0.30%
2-T	7%	0.90%	90.20%	0.10%	1.40%
3-C	2%	8.20%	73%	2.20%	13.20%
3-T	4%	7.10%	62.20%	0.90%	25.40%
4-C	22%	31.10%	25.20%	0.30%	21.10%
4-T	52%	7.80%	28.40%	3.70%	7.60%
5-C	46%	1.60%	43.90%	7.20%	1.50%
5-T	13%	24.20%	49.80%	5.60%	7.10%
6-C	15%	25.10%	32.30%	14.90%	12.40%
6-T	11%	7.90%	75.60%	0.20%	5.50%
7-C	16%	30.90%	40.30%	0.30%	12.40%
7-T	7%	20%	66.50%	1.70%	4.90%
8-C	17%	18.10%	7.10%	0.10%	57.40%
8-T	21%	30.90%	42.60%	2.10%	4%
9-C1	2%	41.50%	21%	3.70%	31.70%
9-C2	3%	39.90%	38.80%	2.10%	16.60%
10-C1	8%	46.70%	28.80%	0.80%	15.90%
10-C2	12%	25.10%	43.40%	12%	6.40%
11-C1	33%	29%	24.10%	1%	12.60%
11-C2	10%	39.50%	36.20%	1.40%	12.30%
12-C1	17%	32.10%	23.70%	20%	7.10%
12-C2	25.60%	49.60%	23.10%	0%	1.70%

Table S3. The relative percentage of various bacterial classes of fecal microbiome in the 8 cats (No. 1-8) of the diarrhea group and the 4 cats (No. 9-12) of the non-diarrhea group before and after the *Bacillus licheniformis*-fermented products (BLFP) treatment. C: before the BLFP treatment; T: after the BLFP treatment; C1: first sampling of feces in the control group; C2: second sampling of feces in the control group.

	Actinobacteria	*Coriobacteriia*	*Bacteroidia*	*Bacilli*	*Clostridia*	*Erysipelotrichia*	*Negativicutes*	*Fusobacteriia*	*Betaproteobacteria*	*Deltaproteobacteria*	*Epsilonproteobacteria*	*Gammaproteobacteria*
1-C	0.3	14.7	24.8	0.3	46.4	1.8	0.1	0.8	2	0.2	6.2	2.4
1-T	0.1	4.1	6.3	0.3	86.3	0.4	1	0.3	0.2	0.1	0	0.6
2-C	0	1.5	0.6	64.1	33	0.1	0.1	0.3	0	0	0	0.2
2-T	0.2	7.1	0.9	1.7	88	0.1	0.4	0.1	0	0	0.1	1.2
3-C	0.2	1.4	7.1	35.9	32.5	2.2	2.4	2.2	5.1	1.6	1	4.4
3-T	0	4.3	7.1	22.7	38.7	0.4	0.4	0.9	0.5	0	0.8	24.1
4-C	0.2	22.2	31.1	0	7.8	1	16.4	0.3	0.2	1.4	4.1	15.3
4-T	20.9	31.2	7.8	1.1	23.2	3	1.1	3.7	0.9	0.1	3.6	2.8
5-C	0	45.8	1.6	0	42.4	0.1	1.4	7.2	0.2	0.3	0	1.1
5-T	0.1	13.2	24.2	0.2	39.9	0.3	9.4	5.6	2.3	0	0	4.7
6-C	0	15.2	25.1	0	27.3	1.3	3.6	14.9	3.3	1	0	8
6-T	0	10.8	7.9	5.6	62.1	6.5	1.4	0.2	2.2	0.1	0.7	2.5
7-C	0.8	15.2	30.9	0	29.5	0.8	10.1	0.3	0.7	0.3	0	11.4
7-T	0	6.8	20	0.1	64	0.6	1.8	1.7	0.2	1.1	0	3.5
8-C	0.5	16.8	18.1	0	4.6	0.1	2.4	0.1	0.2	0	0	57.1
8-T	0	20.4	30.9	0	14.5	0.6	27.5	2.1	0.5	0	0	3.5
9-C1	0	2.1	41.5	0	15.1	0.4	5.4	3.7	1.8	1.6	3.6	24.7
9-C2	0	2.6	39.9	0	35.1	1.4	2.2	2.1	0.8	1.1	4.1	10.5
10-C1	0.1	7.6	46.7	0	8.8	1.1	18.9	0.8	2.1	0.5	2.6	10.7
10-C2	0	12	25.1	0	39.9	3	0.5	13	0.3	0.1	2.5	3.5
11-C1	3.5	29.8	28.9	0.2	13.9	0.2	9.9	1	1.5	0	1	10
11-C2	0.1	10.4	39.5	0	27.8	0.4	8	1.4	0.6	0.2	1.2	10.4
12-C1	0.5	16.4	32.1	0	21.7	0.5	1.6	20	5.3	0.1	0.7	0.9
12-C2	8.6	17	49.6	0	21.2	0.1	1.8	0	0	0	0	1.6

Figure S1. The list of represented bacteria classes of other color bars in Figure 4.

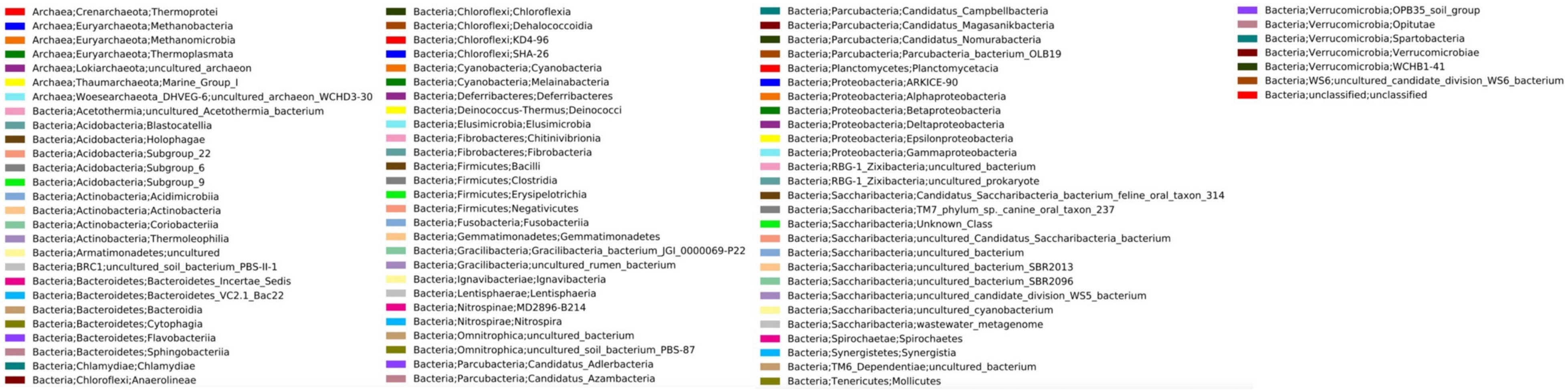

Supplement: Supplementary file 1 [file animals-12-02187-s001.zip › animals-1855078-supplementary.pdf]
